# Identification of phenolics from miracle berry (*Synsepalum dulcificum*) leaf extract and its antiangiogenesis and anticancer activities

**DOI:** 10.3389/fnut.2022.970019

**Published:** 2022-08-15

**Authors:** Fei-Yue Ma, Xiu-Mei Zhang, Ya Li, Ming Zhang, Xing-Hao Tu, Li-Qing Du

**Affiliations:** ^1^South Subtropical Crop Research Institute, Chinese Academy of Tropical Agricultural Science (CATAS), Zhanjiang, China; ^2^Key Laboratory of Tropical Fruit Biology, Ministry of Agriculture, Zhanjiang, China; ^3^Key Laboratory of Hainan Province for Post-Harvest Physiology and Technology of Tropical Horticultural Products, Zhanjiang, China; ^4^Baicheng Academy of Agricultural Sciences, Baicheng, China

**Keywords:** miracle berry, phenolics, zebrafish, MCF-7 xenograft, metabolomics

## Abstract

Miracle berry is well-known for its ability to convert sour foods to sweet. In this study, the secondary metabolites of miracle berry leaves (MBL) were identified by UPLC-DAD-MS, and its antiangiogenesis and anticancer activities were evaluated by using a zebrafish model and the MCF-7 xenograft mouse model, respectively. The result showed that 18 phenolic compounds were identified in MBL extract, and dominated by the derivatives of quercetin and myricetin. The MBL extract showed low toxicity and high antiangiogenesis activity, it significantly inhibited the subintestinal vein vessels development in zebrafish at very low concentration. Furthermore, the MBL extract could promote the apoptosis of tumor cells and significantly inhibit the growth of MCF-7 xenograft tumor. In addition, the analysis of metabolites revealed that the MBL extract inhibited tumor growth by activating the metabolic pathways of unsaturated fatty acids and purines. Overall, this study suggests that MBL extract can be used as a natural anticancer adjuvant in the fields of functional foods.

## Introduction

*Synsepalum dulcificum* (miracle berry) from the family Sapotaceae is known for its ability to convert sour foods to sweet by the glycoprotein miraculin. In addition to protein, lipid, fiber, carbohydrate, vitamin and other nutrients, there are many types of chemical compounds such as alkaloids, saponins, flavonoids, polyphenols, cardiac glycosides and anthraquinones in miracle berry (1, 2). These phenolic antioxidants have been suggested to possess health beneficial functions by inhibiting chronic diseases, such as cardiovascular diseases, obesity, and diabetes (3). Furthermore, miracle berry showed anticancer activity. Wang et al. (4) found that two compounds [(+)-syringaresinol and (+)-epi-syringaresinol] isolated from the stems of miracle berry have cell proliferation inhibition activities on human skin melanoma cells. Seong et al. (5) observed selective cytotoxicities of the extracts from stem and berry of miracle berry, which were cytotoxic in HCT-116 and HT-29 human colon cancer cells, but not in HDFn normal human dermal fibroblasts. Meanwhile, other species of family Sapotaceae also showed anticancer activity. For example, the compounds isolated from the root bark of *Butyrospermum Parkii* showed antiproliferative activity against human breast adenocarcinoma (MDA-MB-231) (6). The ethyl acetate extract of *Argania spinosa* showed cytotoxic activity against human breast cancer cells (MCF-7) (7). However, limited literature is available on clarify the anticancer activity of miracle berry extracts *in vivo*.

Breast cancer is one of the most common malignancies worldwide and the leading cause of cancer death among females (8). Hundreds of new compounds have been approved as anti-cancer drugs since the 1940s when the first drug found to be effective in treating lymphomas. Natural products are an important source of anti-cancer drugs and new drug lead compounds. Natural products and their related drugs account for about 60% of the anti-cancer drugs used currently (9, 10). Paclitaxel (PTX) is one of the most successful natural products used to treat cancers including breast cancer, ovarian cancer and lung cancer. However, its therapeutic efficacy is limited due to drug induced-toxicities and resistance. So, the discovery and development of novel therapeutic drugs for cancer are urgently needed. Cancer cells proliferation mainly depend on surrounding blood vessels to obtain oxygen and nutrients, and tumor metastasis were inseparable from angiogenesis (11). Zebrafish has been widely used as an *in vivo* model for screening the effectiveness of antiangiogenesis or anti-cancer drug (12). Therefore, we aimed to evaluate the anticancer activity of miracle berry leaves (MBL) using a zebrafish model and the MCF-7 xenograft mouse model in this study.

Metabolomics can simultaneously perform qualitative and quantitative analysis of all low molecular weight metabolites of cells, tissues, and biofluids, revealing the overall metabolic response and dynamic changes under different conditions. With the development of mass spectrometry, metabolomics has been used in cancer research to identify biomarkers and perturbed metabolic pathways, investigate the molecular mechanism of carcinogenesis and measure the drug responsiveness (13, 14). In this study, liquid chromatography with mass spectrometer (LC-MS) was applied to identify and quantify the major phenolics in the MBL. On this basis, the capability of MBL against angiogenesis was evaluated by using transgenic zebrafish embryos with fluorescent blood vessels as well. In addition, we established the MCF-7 xenograft mice model to identify potential serum biomarkers of breast cancer and to explore the anticancer mechanisms of MBL extract by metabolomic approach.

## Materials and methods

### Materials and chemicals

HPLC (High Performance Liquid Chromatography) grade acetonitrile, formic acid and methanol were purchased from Fisher Chemicals (Fair Lawn, NJ, USA). The phenolic standards were from Sigma-Aldrich Chemical Company (St. Louis, MO, USA). Fetal bovine serum (FBS), F-12K medium, DMEM (dulbecco's modified eagle medium)/High medium, RPMI (Roswell Park Memorial Institute) 1640 medium and phosphate buffered saline (PBS) were purchased from Invitrogen Company (Carlsbad, CA, USA).

### Preparation of MBL extract

Miracle berry leaves were freeze-dried immediately after being harvested in a local farm during the 2021 in September. The selected plantation is located in Zhanjiang City (longitude: 110°27', latitude: 21°16'), Guangdong Province, which belongs to South Asia Tropical Botanical Garden in China. The dried leaves were ground with a kitchen blender (DFT-200A, 25000 r/min). The leaves powder was mixed with 500 mL of 70% methanol (1:10, w/v), and incubated at 60°C for 2 h in a shaking water bath (SHA-C, 0–300 r/min). Then, the mixture was centrifuged to obtain a supernatant. The supernatant was transferred to a clean flask, and the precipitated residue was mixed with 500 mL of 70% methanol to repeat the extraction and centrifugation operation. The supernatants were combined and dried by a rotary evaporator at 40°C under vacuum. The dried extract was then stored at −20°C before use.

### Identification and quantification of phenolics in MBL extract

The identification and quantification of phenolics were conducted on a Waters UPLC (Ultra Performance Liquid Chromatography, Acquity H-class system, Millford, MA, USA) equipped with a BEH C18 column (50 mm × 2.1 mm × 1.7 μm, Waters) and a diode array detector (DAD) and a Xevo triple-quadruple (TQD) tandem mass spectrometer (MS). MS data were acquired in negative ionization mode for determination of parent ions. The data from DAD and MS detector were collected and processed with Masslynx 4.1 software (Waters, Millford, MA, USA). The maximum absorption wavelength of each compound was used to integrate its peak area. Then, its concentration was calculated based on its calibration curve.

### Analysis of toxicity and antiangiogenesis activity using zebrafish embryo model

The transgenic Tg[fli1:egfp] ^y1^ zebrafish embryos with fluorescent blood vessels were used in this experiment. All experimental procedures in the zebrafish study were based on the American Veterinary Medical Association's (AVMA) Panel on Euthanasia and the Association for Assessment and Accreditation of Laboratory Animal Care (AAALAC) International.

For determination of the toxicity of miracle berry leaf extract, at 24 h post-fertilization (pf), the zebrafish embryos were placed into a 12-well plate with 20 embryos per well. The leaf extract was administered to zebrafish embryos at a series of concentrations. At 72 h post-fertilization (pf), the mortality of each well was accounted and used to calculate the half of lethal concentration (LC_50_) of the leaf extract. For determination of the antiangiogenesis activity of the extract, at 24 h post-fertilization (pf), the embryos were placed into a 12-well plate with 30 embryos per well. Based on the LC_50_ of the leaf extract, 0, 10, 25, or 50 μg/mL of the leaf extract concentration was used to treat zebrafish embryos. After 48 h incubation, the embryos were treated with tricaine and observed for subintestinal veins (SIVs) development by a fluorescence microscope with a digital camera for image records. The area of the SIVs coverage in embryo yolk was quantified by a microscope image analysis software (NIS-Elements D3.1, Tokyo, Japan) to determine the inhibition rate against angiogenesis.

### Analysis of anticancer activity using MCF-7 xenograft mouse model

*In vivo* animal studies were approved by the Ethics Committees of Chinese Academy of Tropical Agricultural Science. All experiments were performed in accordance with relevant guidelines and regulations. MCF-7 cell lines were obtained from the Chinese Academy of Sciences cell library. MCF-7 cells were cultured in DMEM medium supplemented with 10% dialyzed fetal bovine serum (FBS) at 37°C with 5% CO_2_. To establish the tumor xenograft mouse model, MCF-7 cells (4 × 10^7^) were injected subcutaneously into the shoulder of nude mice (BALB/c, 6 weeks old, female). After 2 weeks, the MCF-7 xenograft mice were randomly divided into 4 groups (*n* = 5 per group) for the treatment. Vehicle (negative treated, NT), normal untreated mice (normal control, NC), 20 mg/kg of PTX (positive control), 30 mg/kg of MBL extracts (Low-dose MBL, LSD) and 300 mg/kg of MBL extracts (High-dose MBL, HSD) were intraperitoneal injection into the mice. Tumor size at 1, 3, 5, 9, 14 days after treatment was measured, and the tumor volume was calculated: V = 0.52 × larger diameter × (smaller diameter)^2^. The inhibitory rate was calculated using the tumor volume at the 14 days after treatment as follows: Inhibitory rate (%) = 1-V_d_/${\text{V}}_{\text{n}}^{*}$100. V_d_ is the tumor volume of the drug treatment group and V_n_ is the tumor volume of NT group. Tumor tissue and blood samples were obtained after 14 days of treatment. Blood samples were centrifuged at 2000 g for 5 min followed by centrifugation at 13,500 g for 15 min at 4°C. The serum was aliquot and stored at −80°C until use.

### Tumor cell apoptosis by flow cytometry

Cell apoptosis was detected by annexin V-FITC apoptosis detection kit according to the manufacturer's instructions (BD Biosciences, USA). Briefly, cells (1.5 × 10^5^) were seeded in 6-well plates and were collected after transfection by trypsinization (without EDTA). After being washed with PBS for 2 times, cells were resuspended in 500 μL of 1 × binding buffer, and stained with 5 μL of Annexin V-FITC and 5 μL of propidium iodide (PI, 20 μg/ml) in the dark place at 4°C for 20 min. FITC and PI fluorescence were analyzed by flow cytometry (BD Biosciences, USA).

### GC-MS analysis of serum samples

For each group, serum samples were pooled and derivatized. GC-MS was performed according to a well-established protocol (15). Briefly, 10 μL internal standards (0.3 mg/mL 2-chlorophenylalanine in methanol) were mixed into 80 μL sample. After adding 240 μL mixtures of methanol and acetonitrile (2/1, v/v), the samples were ultrasonicated for 5 min and stand for 10 min at −20°C. After centrifugation at 12,000 rpm for 10 min at 4°C, an aliquot of the 150 μL supernatant was transferred to a glass sampling vial for vacuum-dry at room temperature. And 80 μL of 15 mg/mL methoxylamine hydrochloride in pyridine was subsequently added. The resultant mixture was vortexed for 2 min and incubated at 37°C for 90 min followed by adding 80 μL BSTFA (1% TMCS) and 20 μL *n*-hexane and then derivatized at 70°C for 60 min. Quality Control (QC) sample was prepared by mixing aliquots of all samples to be a pooled sample.

The derivatized samples were analyzed on an Agilent 7890B gas chromatography system coupled to an Agilent 5977A MSD system (Agilent Technologies Inc., CA, USA) with eight technical replicates. A DB-5MS capillary column (30 m × 0.25 mm × 0.25 μm, Agilent) was utilized to separate the derivatives (15). Helium was used as the carrier gas at a constant flow rate of 1 mL/min through the column. The injector temperature was maintained at 260°C. The injection volume was 1 μL in splitless mode. The initial oven temperature was 60°C, ramped to 125°C at a rate of 8°C/min, to 210°C at a rate of 5°C/min, to 270°C at a rate of 10°C/min, to 305°C at a rate of 20°C/min, and finally held at 305°C for 5 min. The temperature of MS quadrupole and ion source (electron impact) was set to 150 and 230°C, respectively. The collision energy was 70 eV. Mass spectrometric data were acquired in a full-scan mode (m/z 50–500). The QCs were injected at regular intervals (every 8 technical repeat samples) throughout the analytical run to provide a set of data from which repeatability could be assessed.

Analysis Base File Converter software was used to convert the raw data (.D format) to abf format. Then the data was imported into the MS-DIAL software for data processing. Metabolites were annotated through the Fiehn database. All internal standards and any known pseudo positive peaks were removed. Missing values were replaced with 0. The data were normalized and the peaks from the same metabolite were combined (16).

### Statistical analysis

SIMCA software package (14.0, Umetrics, Umeå, Sweden) was used for statistical analysis of metabolomics data with PCA (principal component analysis) and OPLS-DA (orthogonal projections to latent structures discriminant analysis). The differential metabolites were selected based on the combination of a statistically significant threshold of variable influence on projection (VIP) values obtained from the OPLS-DA model and *p*-values from a two-tailed Student's *t*-test on the normalized peak areas from different groups, where metabolites with VIP values larger than 1.0 and *p*-values <0.05 were considered as differential metabolites. Kyoto Encyclopedia of Genes and Genomes (KEGG) enrichment analysis was performed with MBRole (17). For other results, the SPSS software package (Version 16.0) was used for statistical analysis with unpaired Student's *t*-test. The data are presented as means ± SD. *p* < 0.05 was defined as statistical significance.

## Results and discussion

### Phenolics in MBL extract

**Figure 1:**
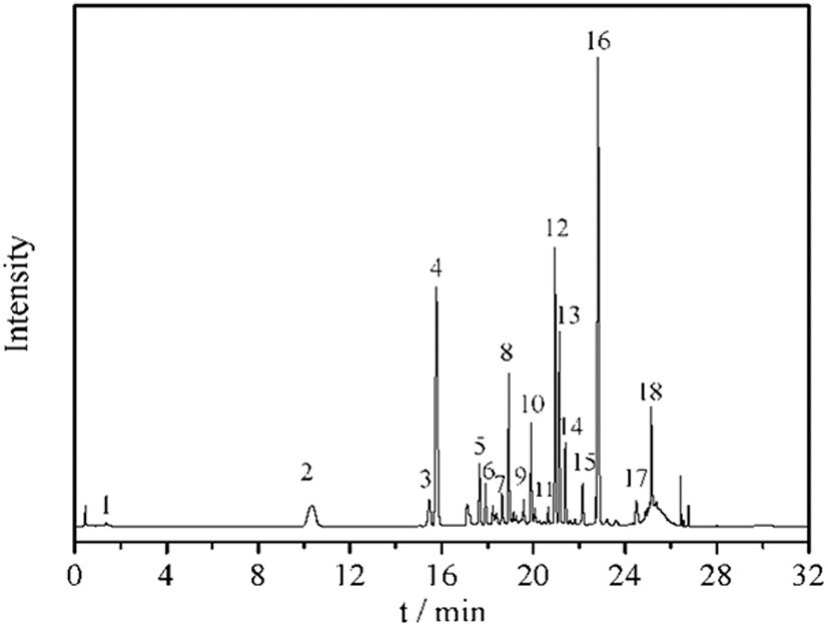
Chromatogram and UV and MS spectrum data of phenolics in miracle berry leaf extract. 1. gallic acid; 2. 5-caffeoylquinic acid; 3. 5-O-p-coumaroylquinic acid; 4. 3-O-p-coumaroylquinic acid; 5. 3-O-feruloylquinic acid; 6. 4-O-caffeoylquinic acid; 7. 4-O-p-coumaroylquinic acid dimer; 8. 4-O-p-coumaroylquinic acid; 9. 4-O-feruloylquinic acid; 10. myricetin-3-galactoside; 11. rutin; 12. myricetin-3-O-rhamnoside; 13. quercetin-3-D-galactoside; 14. quercetin-3-glucoside; 15. kaempferol-3-O-glucoside; 16. quercetin-3-rhamnoside; 17. myricetin-O-galloyl rhamnoside; 18. quercetin 3-O-α-(2”-galloyl) rhamnoside.

Eighteen different phenolic phytochemicals were identified in the miracle berry leaf extract (Figure 1). Among them, 3-O-p-coumaroylquinic acid, myricetin-3-O-rhamnoside, quercetin-3-D-galactoside and quercetin-3-rhamnoside are the dominant phenolics in the extract. Their concentrations were from 3.0 mg/g of myricetin-3-O-rhamnoside to 12.2 mg/g of quercetin-3-rhamnoside. Five phenolic acids, p-hydroxybenzoic acid, vanillic acid, syringic acid, trans-p-coumaric acid, and veratric acid, were identified in the aqueous extract of miracle berry leaves at a level of below 3 mg/g.

The level of quercetin derivatives in the MBL extract was much higher than that in other berry fruit leaves such as blackberry leaves (7.0 mg/g), bilberry leaves (4.6 mg/g) and black currant leaves (2.6–4.2 mg/g), while the myricetin derivatives level was higher than black currant leaves (0.061–0.078 mg/g) and blueberry leaves (0.056–0.141 mg/g) (18). Some reports confirmed that these berries leave extracts also have antioxidant and anti-tumor properties (19). The profile of phenolics in the miracle berry leaves was different from that in miracle berry flesh. Only 12 phenolics were found in miracle berry flesh with epicatechin and malvidin galactoside as the major phenolics (12). The phenolics found in the MBL have been suggested to associate with health benefits by many studies (20, 21). For example, quercetin derivatives exhibited antioxidant, anticancer and neuroprotective therapeutic potentials (22).

### Toxicity and antiangiogenesis activity of miracle berry leaf extract

**Figure 2:**
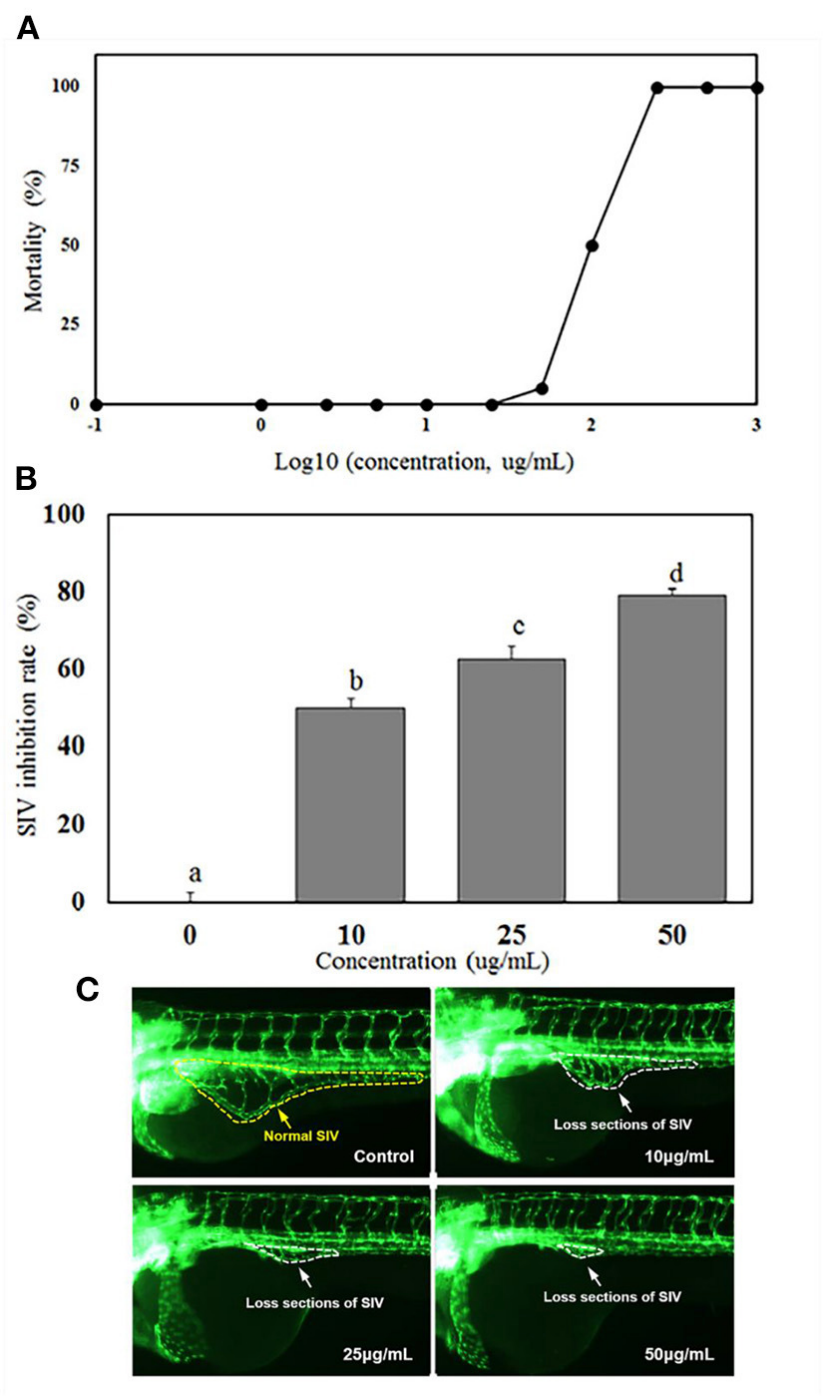
Mortalities of zebrafish embryo at different concentrations of MBL extract **(A)**, inhibition rates of subintestinal vein (SIV) developments **(B)**, and fluorescent images of zebrafish embryos **(C)** at different MBL extract concentrations. Bars with different letters indicate a significant difference (*p* < 0.05).

Figure 2A showed 0% mortality of zebrafish at the extract concentration <25 μg/mL, while the LC_50_ (lethal dose, 50%) of the extract to zebrafish was 100 μg/mL. Zebrafish is a vertebrate system which has been widely used as a high-throughput model of angiogenesis for drug screening. Generally, the subintestinal vein vessels (SIVs) of zebrafish originate from the duct of Cuvier area and gradually form a vascular plexus across most of the dorsal-lateral aspect of the yolk ball. As shown in Figures 2B,C, normal SIVs development was observed in the control group which the SIVs formed an integral net-like structure. However, abnormal development of SIV were observed in all treatment groups (Figure 2C). In the group treated with 10 μg/mL of MBL extract, the inhibition rate of SIV formations was 50.05% and increased to 62.78 and 79.21% at the extract concentrations of 25 and 50 μg/mL, respectively (Figure 2B).

The LC_50_ level of miracle berry leaf extract ( ≤ 100 μg/mL) was significantly higher than most of other plant or herb extracts reported in previous studies (23–25). For example, curcumin, a safe polyphenol possessing antioxidant activity and health benefit in prevention of different chronic diseases, had mortality of zebrafish at 2.76 μg/mL (23). The extract of *Millettia pachycarpa* Benth (a Japanese traditional medicine) was rich in bioactive flavonoids with anti-inflammatory function, and had a LC_50_ as low as 3 μg/mL to zebrafish (24). The *Cinnamon zeylanicum* and *Eugenia polyantha* extracts, which are popular and safe supplements for health promoting, reached to 50% of mortality (LC_50_) at concentration of 50.6 and 60.4 μg/mL, respectively (25). Therefore, compared with those plant extracts, the miracle berry leaf extract had the higher LC_50_ and lower toxicity.

It was demonstrated that the MBL extract had an effective inhibitory effect on angiogenesis in zebrafish. Similar results have been reported that some herbal extracts. *Kaempferia galanga* L. and *Inula helianthus-aquatica* exhibited the antiangiogenesis activities in zebrafish model (26, 27). Red wine extract significantly inhibited the formation of intersegmental vessel (ISV) with incomplete structure and occasional sprouts (28). It has been confirmed that the antioxidant activity of phenolics in those plant extracts and red wine mainly contributed to the antiangiogenesis capability (28). As the MBL extract was rich in the derivatives of quercetin and myricetin, these antioxidant phenolics could mainly contribute to the inhibition activity against angiogenesis in zebrafish.

### Effects on the tumor growth of MCF-7 xenograft mice

**Figure 3:**
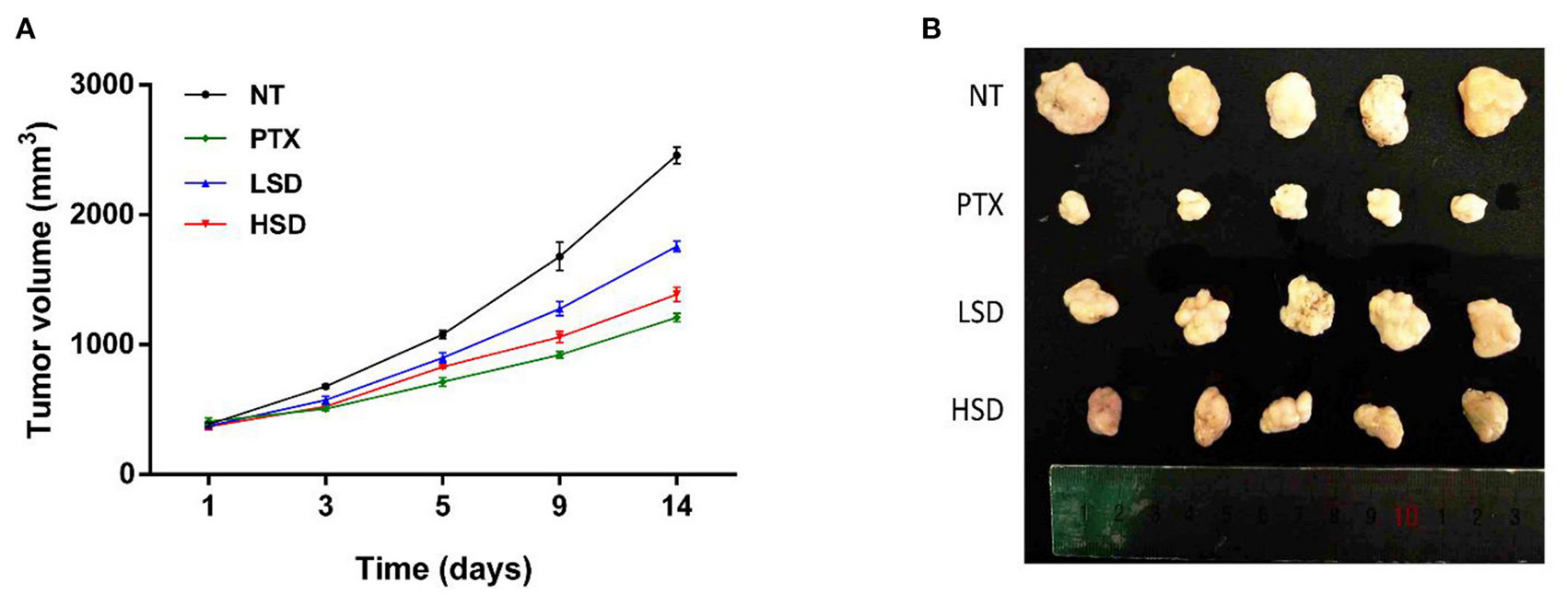
Effect of PTX and MBL extract on the growth of MCF-7 tumors. **(A)** Growth curves of MCF-7 xenograft tumors from different treated groups after treatment. **(B)** Photographs of all xenograft tumors in mice.

MCF-7 xenograft mice was established by subcutaneous injections of MCF-7 cells into the shoulder of female nude mice. After the treatment of PTX, LSD and HSD, the tumor size of MCF-7 xenograft mice was measured within 14 days. As shown in Figure 3A, the tumor volume in PTX, LSD and HSD group was smaller than that in the NT group after 3 days of treatment. PTX group showed the smallest tumor volume after 5 days of treatment, while the tumor volume in the LSD group and HSD group had obvious difference after 9 days. At the end of 14 days of treatment, the mean tumor volumes were significantly lower (*p* < 0.01) in PTX, LSD and HSD group compared with that in NT group (Figure 3B). The mean inhibitory rates were 50.79, 28.56, and 43.64% in PTX, LSD, and HSD group, respectively.

As a widely used anticancer drug, PTX showed the best anticancer activity in MCF-7 xenograft mice in our study. However, the resistance of breast cancer to PTX treatment (29) and side effects of PTX such as hypersensitivity, neuropathies and cardiotoxicities (30, 31) are the obstacles in clinical application. Therefore, the study of new plant-derived anticancer drugs such as MBL extract is valuable for breast cancer therapy. Our results showed that the MBL extract significantly inhibits the growth of tumor of MCF-7 xenograft mice in a dose-dependent manner. This result may be related to the presence of gallic acid, chlorogenic acid, rutin and oleanolic acid in the MBL extract (15). Many studies (16, 32, 33) have shown that the natural phenolic compounds such as gallic acid and chlorogenic acid has antitumor effects on breast cancer. In addition, the flavonoids rutin (34) and oleanolic acid (35) have also been reported to have antitumor effects on breast cancer.

### Effect on tumor cell apoptosis

**Figure 4:**
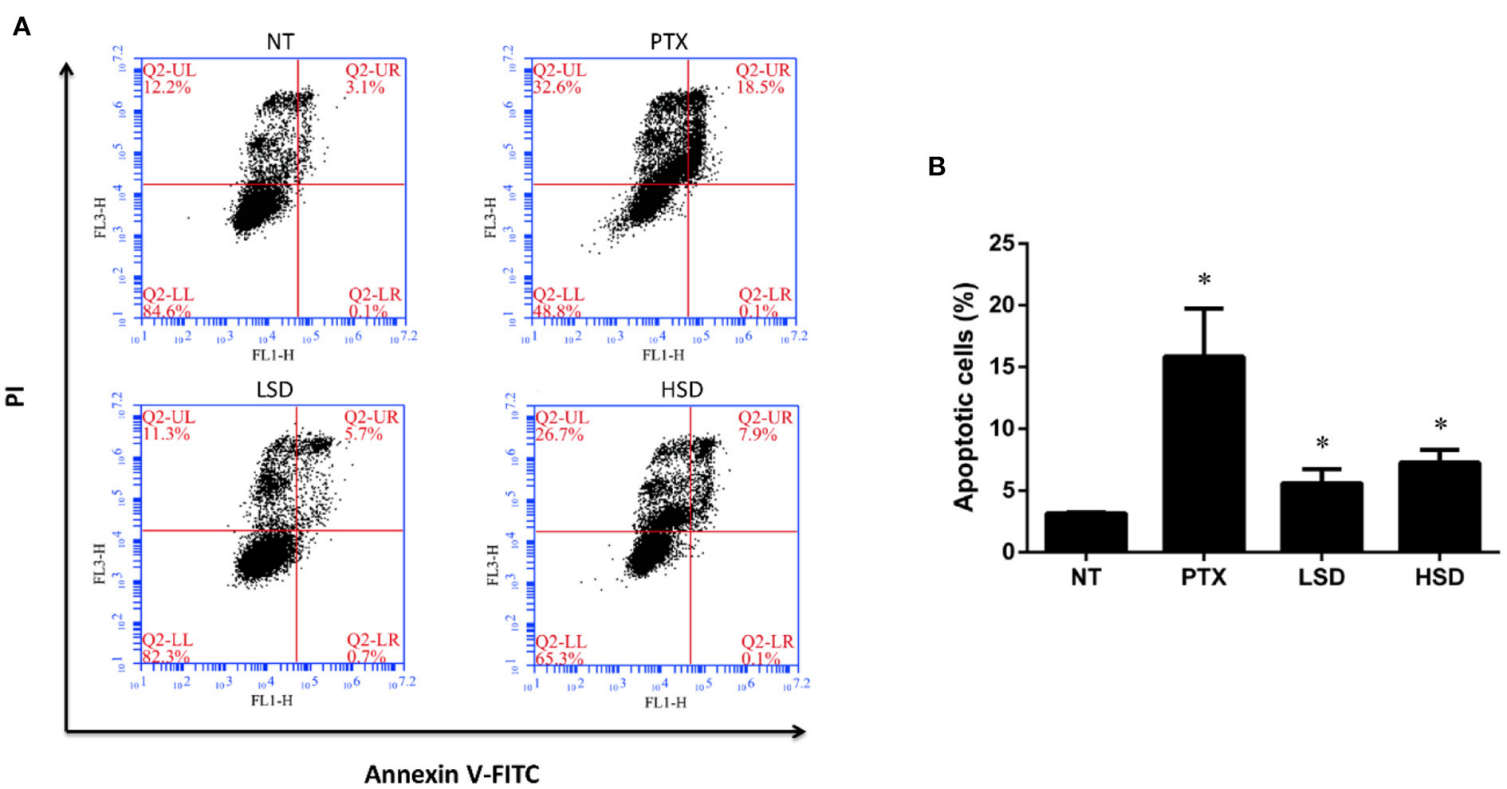
Apoptosis of tumor cells induced by PTX and MBL extract was detected by flow cytometry. **(A)** Tumor cells were stained with Annexin V-FITC/PI for flow cytometry analysis. Apoptotic cells were divided into 2 stages (LR for early apoptotic cells and UR for late apoptotic cells). **(B)** The total apoptotic rates examined by flow cytometry, including the early and late apoptosis. *p < 0.05.

To examine the role of PTX and MBL extract on the inhibition of tumor growth, flow cytometry was used to detect the apoptosis of tumor cells in different groups. As shown in Figure 4A, FCM showed that the percentage of apoptotic cells (including the early and late apoptosis) was highest in the PTX group, followed by HSD group and LSD group, which were all significantly elevated (*p* < 0.05) compared to NT group (Figure 4B). This result indicated that PTX and MBL extract treatment could promote the apoptosis of tumor cells in MCF-7 xenograft mice.

It is reported that the anticancer mechanism of PTX leading to the stabilization of microtubule, cell arrest, and apoptosis (36). Pan et al. (37) reported that PTX could induce apoptosis in breast cancer cells. In our study, we observed increased tumor cell apoptosis in PTX treated group as well as MBL extract treated groups with flow cytometry, which indicate that the anticancer mechanism of MBL extract involves the induction of tumor cell apoptosis. Gallic acid, chlorogenic acid, rutin and oleanolic acid in the leaf extract (15) may induce breast cancer cell apoptosis. For example, Moghtaderi et al. (38) showed that gallic acid could induce apoptosis in human breast cancer cell. Zeng et al. (39) showed that chlorogenic acid induces apoptosis in breast cancer via the NF-κB signaling pathway. Saleh et al. (40) reported the anticancer activity of rutin in MCF-7 cell line. Chu et al. (35) reported that oleanolic acid derivative SZC014 induced apoptosis of human breast cancer cells.

### Metabolic biomarkers of MCF-7 xenograft

**Figure 5:**
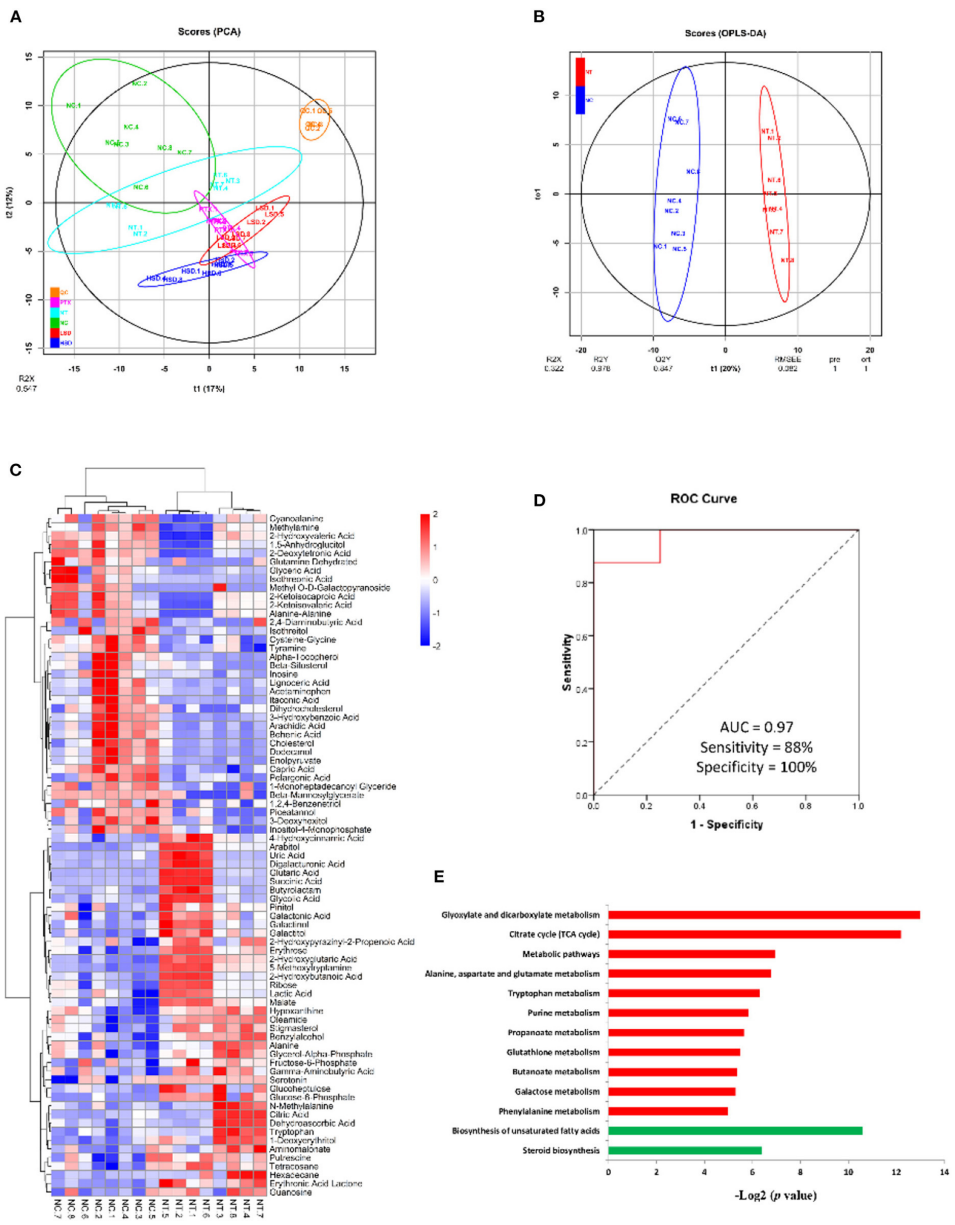
Metabolomics analysis of MCF-7 xenograft mice serum. **(A)** PCA scores of serum metabolites from NC, NT, PTX, LSD, and HSD group. **(B)** OPLS-DA scores plots of MCF-7 xenograft mice and normal control. **(C)** Hierarchical clustering of serum metabolome. The heat map represented the Z scores of significantly differentially expressed metabolites between the NC and the NT group. **(D)** ROC curves for the combination of serum succinic acid and glucose-6-phosphate to discriminate MCF-7 xenograft mice from normal control. **(E)** Enriched KEGG pathway in 42 up-regulated (red) and 37 down-regulated (green) metabolites of NT group compared to the NC group. The x axis shows the enrichment significance presented with –log2 (*P*-value).

To identify biomarkers of breast cancer and investigate the effect of drug treatment on the metabolism of MCF-7 xenograft, we used gas chromatography mass spectrometry (GC-MS)-based metabolomics approach to untargeted identify and quantify the metabolites in NC, NT, LSD, HSD, and PTX group. In total, 257 metabolites were identified and quantified in all groups (Supplementary table 1). PCA analysis showed that QC samples are gathered together (Figure 5A), indicating the good stability of the mass spectrometry results. The result of PCA and OPLS-DA scores (Figure 5B) plots indicated a clear separation between the NC and the NT group. Seventy-nine significantly differentially expressed metabolites (DEMs) were identified based on univariate and multivariate statistical methods (*p* < 0.05, VIP > 1) (Figure 5C). Among those DEMs, succinic acid and glucose-6-phosphate had the highest fold changes between NT and NC group, which may the potential biomarkers for breast cancer. The combination of serum succinic acid and glucose-6-phosphate could distinguish MCF-7 xenograft mice (AUC = 0.97, sensitivity = 88%, specificity = 100%) from normal controls (Figure 5D). Although the number of samples in our study was very few and the results need further verification, this preliminary result showed that serum metabolites have the potential to become biomarkers for breast cancer.

To identify biological function changes in MCF-7 xenograft mice, metabolite names of DEMs were converted to KEGG ID to perform KEGG enrichment analysis. The significantly enriched KEGG pathways were shown in Figure 5E. Among the 42 up-regulated metabolites in the MCF-7 xenograft group, pathways including glyoxylate and dicarboxylate metabolism, TCA cycle, alanine-aspartate and glutamate metabolism, tryptophan metabolism, purine metabolism, propanoate metabolism, glutathione metabolism, butanoate metabolism, galactose metabolism and phenylalanine metabolism were enriched. While biosynthesis of unsaturated fatty acids and steroid biosynthesis pathways were significantly enriched in the 37 down-regulated proteins.

### Metabolite variations in PTX, LSD, and HSD treated MCF-7 xenograft mice

**Figure 6:**
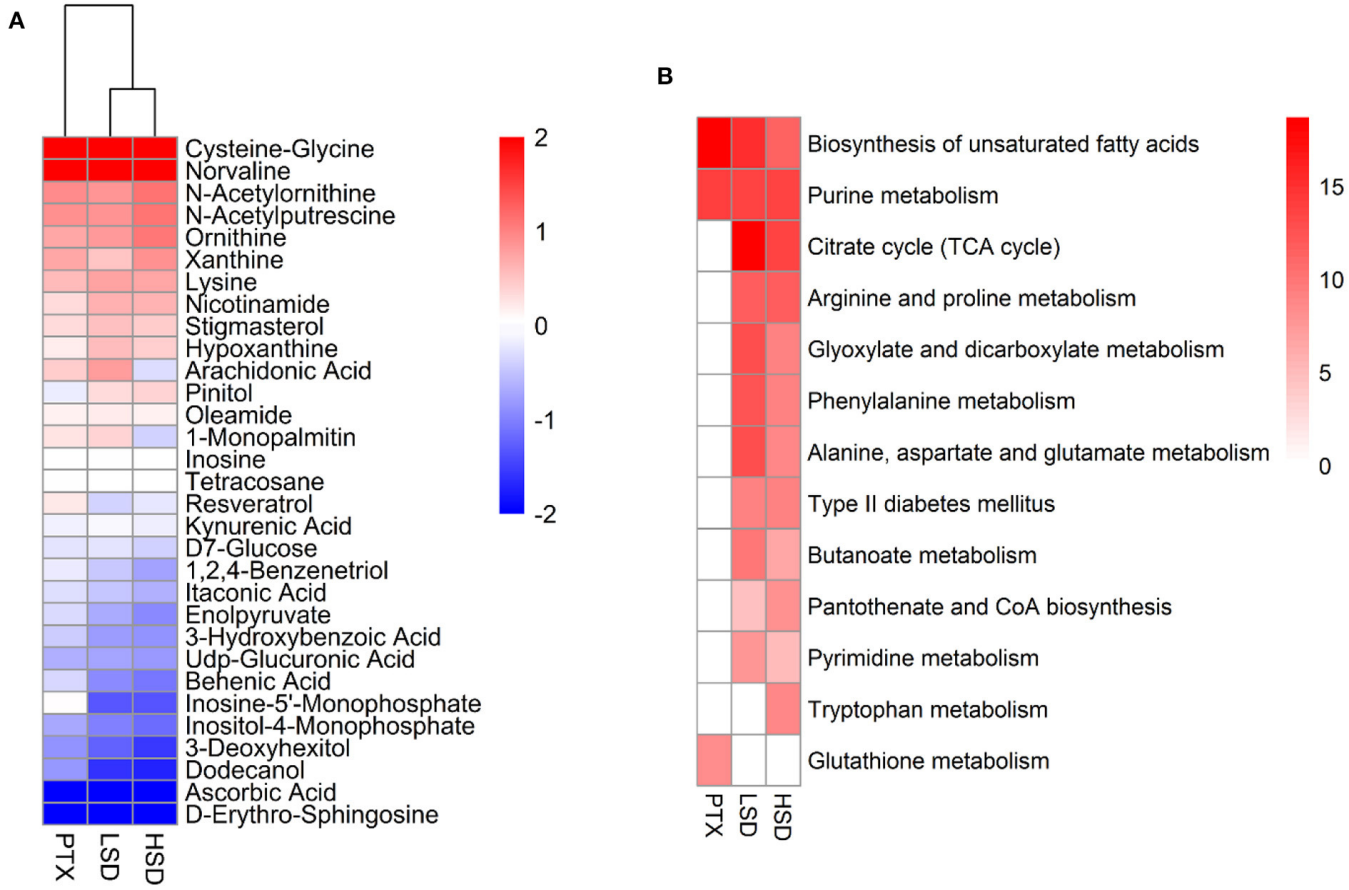
Metabolomics analysis of PTX, LSD, and HSD treated MCF-7 xenograft mice. **(A)** Heatmap shows the commonly changed metabolites in the treatment groups compared to the NT group. The data were represented as the log2 of fold change (treatment/NT). **(B)** The enriched metabolic pathways in PTX, LSD and HSD groups compared to NT group. The heat map represented –log2 (*P*-value) of enrichment significance. White square means pathway term was not enriched.

The PCA result showed a clear separation between the treatment group and the NT group (Figure 5A). After comparing the treatment group and the NT group with OPLS-DA, 70, 101, and 96 significantly DEMs (*p* < 0.05, VIP > 1) were identified in PTX, LSD and HSD, respectively. There are 30 metabolites that commonly changed in the treatment groups compared to the NT group (Figure 6A). Among them, cysteine-glycine and norvaline had the highest upregulation in all treatment groups, while ascorbic acid and d-erythro-sphingosine were the two DEMs with the largest down-regulated levels in all treatment groups. KEGG enrichment analysis was performed to identify the changing metabolic pathways for each treatment group. The enriched metabolic pathways in PTX, LSD, and HSD groups, when compared to the NT group, were shown in Figure 6B. Biosynthesis of unsaturated fatty acids and purine metabolism were enriched in all treatment groups. Nine pathways including TCA cycle, arginine and proline metabolism, glyoxylate and dicarboxylate metabolism, phenylalanine metabolism, alanine-aspartate and glutamate metabolism, type II diabetes mellitus, butanoate metabolism, pantothenate and CoA biosynthesis and pyrimidine metabolism were enriched in both LSD and HSD group. While tryptophan metabolism was enriched in the HSD group and glutathione metabolism was enriched in the PTX group. The different DEMs and enriched metabolic pathways in treatment groups when compared to the NT group indicate that PTX and MBL extract have a different mechanism of action. And the mechanism of MBL extract may depend on the concentration of the drug, which is similar to PTX (41).

### Co-regulated metabolic pathways in PTX, LSD, and HSD treated MCF-7 xenograft mice

**Figure 7:**
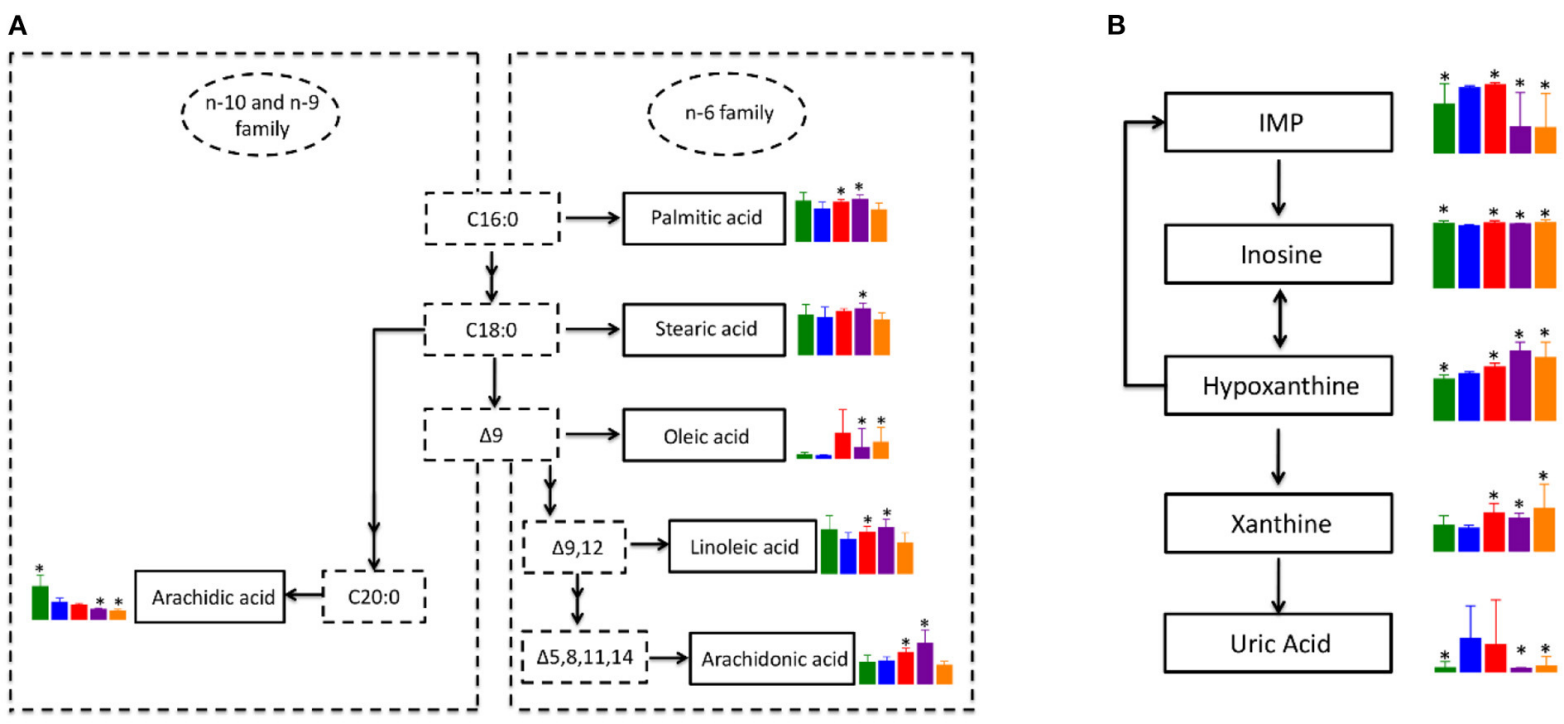
Co-regulated metabolic pathways in PTX and MBL extract treated MCF-7 xenograft mice. **(A)** Altered unsaturated fatty acids metabolic pathway. **(B)** Altered purine metabolism. Boxes represented for the level of metabolites identified in NC (green), NT (blue), PTX (red), LSD (purple), and HSD (orange) group. Data represent means ± standard deviation. * is represent statistical significance with *P* < 0.05 when compared to NT group.

Since biosynthesis of unsaturated fatty acids and purine metabolism pathways were significantly enriched in all treatment groups when compared to the NT group, we focus on those metabolic pathways for further analysis. Although not significant, blood levels of saturated fatty acids in the n-6 family, including palmitic acid and stearic acid were down-regulated in NT group compared to the NC group (Figure 7A). While the level of palmitic acid and stearic acid were significantly up-regulated in PTX and LSD groups when compared to the NT group. Oleic acid, which is classified as a monounsaturated fatty acid, was elevated in all three treatment groups when compared to the NT group. The level change of polyunsaturated fatty acid in the n-6 family, including linoleic acid and arachidonic acid, were similar to saturated fatty acids of the n-6 family. However, the blood level of arachidic acid, which is a saturated fatty acid in the n-10 and n-9 family, was significantly down-regulated in the NT group and was further reduced in all treatment groups compared to the NC group. Overall, the biosynthesis of fatty acids in the n-6 family was inhibited in MCF-7 xenograft mice, but was recovered or even further activated in PTX and LSD treated group. While the fatty acid synthesis in the n-9 and n-10 family was inhibited in MCF-7 xenograft mice and all treatment groups, especially in MBL extract treated groups. Like above mentioned, Peng et al. (42) observed decreased unsaturated fatty acids metabolism in MCF-7 xenograft mice, which is consistent with our result. Accumulated evidence has demonstrated that n-6 unsaturated fatty acids such as linoleic acid (43, 44) and arachidonic acid (45, 46) have an important role in cancer cell growth and apoptosis. Therefore, we think that the increased unsaturated fatty acids in PTX and MBL extract treated group may help induce tumor cell apoptosis.

The pathway of purine metabolism is complex and involves many enzymes and metabolites. However, the DEMs of the purine metabolism pathway identified in our study are mainly involved in xanthine metabolism. As shown in Figure 7B, inosine monophosphate (IMP) is converted into inosine, which could be interconverted with hypoxanthine. Hypoxanthine could also convert into IMP by hypoxanthine-guanine phosphoribosyltransferase. Xanthine is formed from oxidation of hypoxanthine, which is catalyzed by xanthine oxidase to product uric acid. In MCF-7 xenograft mice, the level of hypoxanthine and uric acid were significantly up-regulated when compared to normal control. While the level of xanthine had no significant difference between NT and NC group, indicating the consumption of xanthine to produce more uric acid in breast cancer. In the LSD and HSD treated group, the level of IMP and uric acid was significantly lower than that in the NT group. But the expression of inosine, hypoxanthine and xanthine was higher in MBL extract treated groups than that in the NT group. Some studies reported that gallic acid (47) and rutin (48) could inhibits xanthine oxidase, which was consistent with our results. The inhibitory effect of these components in the MBL extract on xanthine oxidase may also be related to their antitumor effect (49). The increased production and the decreased consumption indicate the accumulation of xanthine in the MBL extract treated groups. Our results for the first time demonstrated that xanthine was accumulated in MBL extract treated MCF-7 xenograft mice, which may be a biomarker for monitoring drug efficacy. In addition, the level change of metabolites in the purine metabolism pathway in the PTX group was consist with MBL extract treated groups except for IMP and uric acid. The level of uric acid in the PTX group had no significant difference with that in the NT group, indicating the activation of the purine metabolism pathway in the PTX group.

## Conclusions

This study revealed that miracle berry leaf was rich in phenolic phytochemicals, especially quercetin and myricetin derivatives. The potential of the extract against cancer development of the extract is confirmed by its suppressing activity on the angiogenesis in zebrafish. Metabolomics technology identified new potential serum biomarkers for breast cancer. Pathway analysis showed that the increased unsaturated fatty acids and the accumulation of xanthine in MBL extract treated groups may be beneficial in suppressing tumor growth. This study showed that the MBL extract has the potential to be an anticancer drug for breast cancer.

## Data availability statement

Publicly available datasets were analyzed in this study. This data can be found here: The mass spectrometry data have been deposited to the MetaboLights (https://www.ebi.ac.uk/metabolights/) with the data set identifier MTBLS1897 (https://www.ebi.ac.uk/metabolights/reviewerca47b4c9-4bf8-4b49-a654-4225df2659ba).

## Ethics statement

The animal study was reviewed and approved by Chinese Academy of Tropical Agricultural Science (CATAS).

## Author contributions

L-QD: conceptualization, writing—review and editing, project administration, and funding acquisition. F-YM and X-MZ: methodology. F-YM: software, data curation, writing—original draft preparation, and visualization. YL: writing—review. MZ and YL: validation. X-HT: resources. All authors have read and agreed to the published version of the manuscript.

## Funding

This research was funded by the Fund on Basic Scientific Research Project of Nonprofit Central Research Institutions (Grant Number 1630062021012), the Innovation Team of Modern Agricultural Industry Technology System in Guangdong Province of China (Grant Number 2021KJ116), and the Natural Science Foundation of Hainan province (Grant Number 320QN320).

## Conflict of interest

The authors declare that the research was conducted in the absence of any commercial or financial relationships that could be construed as a potential conflict of interest.

## Publisher's note

All claims expressed in this article are solely those of the authors and do not necessarily represent those of their affiliated organizations, or those of the publisher, the editors and the reviewers. Any product that may be evaluated in this article, or claim that may be made by its manufacturer, is not guaranteed or endorsed by the publisher.
